# The Journalists Initiatives on Immunisation Against Polio and Improved Acceptance of the Polio Vaccine in Northern Nigeria 2007–2015

**DOI:** 10.1093/infdis/jiv545

**Published:** 2016-04-02

**Authors:** Charity Warigon, Pascal Mkanda, Richard Banda, Furera Zakari, Eunice Damisa, Audu Idowu, Samuel Bawa, Emmanuel Gali, Sisay G. Tegegne, Kulchumi Hammanyero, Peter Nsubuga, Charles Korir, Rui G. Vaz

**Affiliations:** 1 World Health Organization, Country Representative Office; 2 National Primary Health Care Development Agency, Abuja, Nigeria; 3 World Health Organization, Regional Office for Africa, Brazzaville, Congo; 4 Global Public Health Solutions, Atlanta, Georgia

**Keywords:** Journalists Initiative on Immunization Against Polio, strong resistance to immunization, noncompliance, partnership

## Abstract

***Background.*** The polio eradication initiative had major setbacks in 2003 and 2007 due to media campaigns in which renowned scholars and Islamic clerics criticized polio vaccines. The World Health Organization (WHO) partnered with journalists in 2007 to form the Journalists Initiatives on Immunisation Against Polio (JAP), to develop communication initiatives aimed at highlighting polio eradication activities and the importance of immunization in northern Nigeria.

***Methods.*** We evaluated the impact of JAP activities in Kaduna State by determining the total number of media materials produced and the number of newspaper clips and bulletins published in support of polio eradication. We also determined the number of households in noncompliant communities that became compliant with vaccination during 2015 supplementary immunization activities (SIAs) after JAP interventions and compared caregivers’ sources of information about SIAs in 2007 before and after the JAP was formed.

***Results.*** Since creation of the JAP, >500 reports have been published and aired, with most portraying polio vaccine positively. During June 2015 SIAs in high-risk wards of Kaduna STATE, JAP interventions resulted in vaccination of 5122 of 5991 children (85.5%) from noncompliant households. During early 2007, the number of caregivers who had heard about SIA rounds from the media increased from 26% in January, before the JAP was formed, to 33% in March, after the initiation of JAP activities.

***Conclusions.*** The formation of the JAP resulted in measurable improvement in the acceptance of polio vaccine in northern Nigeria.

Nigeria, which is among the countries that have never interrupted transmission of indigenous poliovirus, experienced a huge resurgence of polio cases in 2004, after the cessation of polio campaigns in some states in the northern part of the country because of religious misconceptions that oral polio vaccine (OPV) could contain antifertility drugs and human immunodeficiency virus (HIV) [1–7].

Negative campaigns against polio vaccine in the media contributed to the high level of refusals by caregivers, resulting in high numbers of unimmunized children. These negative campaigns were initiated by scholars and religious leaders, with the various media platforms used as the tool for persuading the audience who were largely nonliterate to belief that vaccines were contaminated [8–12].

The delayed immunization of children resulted in the spread of new poliovirus infections in Nigeria and, allegedly, to other parts of western and central Africa, jeopardizing previous accomplishments of the global campaign. By the end of 2006, Nigeria had 1124 children with polio due to wild poliovirus, which was 56% of the global burden (1998 cases). In the same year, owing to weak routine immunization performance, circulating vaccine-derived poliovirus emerged in Nigeria, where 22 of 26 cases (85%) globally were detected [13, 14].

The World Health Organization (WHO), seeing that journalists were a major group in the spreading the negative campaign in various media, decided to partner with them to counter the further spread of the negative messages. Pursuant to achieving the overall goal of polio eradication and after several consultations and sensitization of an initial group of journalists, the Journalists Initiatives on Immunisation Against Polio (JAP) was formed in 2007 by Nigerian journalists working for international, national, and local media companies to carry out communication initiatives aimed at highlighting polio eradication activities and the importance of immunization in northern Nigeria [15].

Previous studies of journalists' involvement in public health concentrated on their media advocacy roles. The studies tended to enunciate the social responsibility of journalists, which only highlighted news reportage and media coverage activities conducted by the journalists for creating awareness on public health [16–19]. This report highlights the broader role played by the JAP following the partnership with the WHO and, later, with the Nigerian government and the United Nations Children's Fund (UNICEF) to portray the association as a critical change agent, instrumental to getting endorsements and statements from key religious and political leaders and helpful in changing the landscape to allow for more penetration among hitherto hard-to-convince populations. As documented, the JAP not only covered and monitored immunization activities in the media, but also sensitized and educated the grassroots communities, especially parents and guardians in noncompliant communities, about immunization activities.

## METHODS

### Setting Up the JAP

In northern Nigeria, polio vaccine noncompliance was allegedly associated with myths propagated by scholars and clerics in the media in Kaduna State that the vaccine contained antifertility organisms and was a western or American ploy to stop the expansion of the Muslim population. At the beginning of the partnership, journalists were mapped to categorize them into print and electronic media because both the print and electronic media from Kaduna State inform individuals in northern Nigeria and are very much part of the national media. The local team in Kaduna state selected journalist on the basis of information about their possible influence in communities that were resisting vaccination activities. The following 8 journalists were identified as the most influential and termed “friends of polio immunization,” based on their positive reports and interest over the years: Nura Mohammed (BBC Hausa service), Ibrahim Kaalmasi Garba (VOA Hausa service), Kunle Sanni (New Nigerian Newspapers), Ahmed Maiyaki (Radio France Internationale), Ibrahima Yakubu (Deutche Welle), Abdulraheem Auodu (Spectator), Modupe Ayoola (News Agency of Nigeria), and Shannar Nyam (Africa Independent Television).

After the initial meeting, these 8 journalists were sensitized with the support of the WHO on the status of polio eradication, the number of children paralyzed, and the negative effects of anti–polio vaccine campaigns in the media. Given the urgency of the situation and the role expected from the pioneer members, they offered to form a body within the Nigerian Union of Journalists that is dedicated to spreading positive messages about polio. Their objectives included creating public awareness on the Polio Eradication Initiative (PEI) through the provision of accurate and balanced information; mobilizing fellow journalists against negative press regarding polio vaccination; mobilizing the support of religious leaders, traditional leaders, and other stakeholders to address noncompliance to immunization; monitoring and evaluating immunization exercises in Nigeria at all times; and providing feedback to communities and policy makers on progress and remaining challenges. The WHO offered to provide technical support and initial funding for the JAP to further organize enlightenment activities, first with their peers on immunization and then in the communities that resisted the polio vaccine.

### What the JAP Did for the PEI

#### Network of Journalists

The instant success with the JAP in Kaduna provided the gateway to expand to other states in northern Nigeria witnessing polio resurgence due to noncompliance. From 2009 to 2010, the JAP inaugurated state chapters in Kaduna, Kano, Jigawa, Sokoto, Niger, the Federal Capital Territory (FCT), Bauchi, Borno, Zamfara, and Katsina. It resolved to expand in 2009 to other northern states where there were urgent needs for its activities, with the aim of building a network of journalists to support the overarching PEI goals.

#### Advocacy for Religious Leaders’ Support

The JAP conducted a series of advocacy meetings and was credited with mobilizing the support of religious leaders for polio immunization, whenever an anti-OPV campaign was launched. Renowned sect leaders, such as Sheik Dahiru Bauchi, Ibrahim EL-Zazzaky, Sheik Mahmud Ahmed Gummi and Dr Khalid Aliyu, the Secretary General of the Jamatul Nasrul Islam, the umbrella organization for Muslims in Nigeria, endorsed the polio vaccine following advocacy meetings with the JAP. The JAP addressed the northern governors forum, which informed the formation of Governors Against Polio and assisted in the mobilization of resources for polio campaigns in the north.

##### Town Hall Meetings and Local Theater for Social Mobilization

The JAP facilitated the conduct of >100 town hall meetings and performances in local drama theaters in noncompliant communities, which led to the improvement of the quality of supplementary immunization activities (SIAs) in communities with strong resistance to immunization. The town hall meetings were systematic, planned, and followed an evidence-based process seeking to promote positive and measurable individual and group behavior and attitudinal change in noncompliant communities. The town hall meetings also mobilized community leaders and provided a forum for discourse between the community and service providers and were moderated by the JAP members with the aim of reaching a positive compromise. Town hall meetings were conducted prior to, during, or after the immunization campaign. The JAP and community influencers used the line list of noncompliance to invite the heads of noncompliant households, youth leaders, and other members in the communities to a meeting at the traditional or religious leader's house to discuss the benefits of immunization and risk of continuous noncompliance.

##### Media Breakfast Meetings and Coverage for Mass Communication

The JAP was able to mobilize free airtime and resources from reputable media companies for polio immunization coverage, despite their commercial drive. Africa Independent Television, News Agency of Nigeria, Kaduna State Media Corporation, Nagarta Radio, DITV, Nigerian Television Authority, New Nigerian, Leadership, Daily Trust, Nigerian Newsday, Freedom Newspaper, Nigerian Tribune, BBC-Hausa, VOA-Hausa, DW-Hausa, and RFI-Hausa were some of the media companies that supported JAP at the initial stage, but others have contributed in equal measure across the northern states. The immunization messages as well for resources mobilized were germane to creating awareness and visibility for SIAs. The JAP also supported local government areas (LGAs) to cover public launch of supplemental immunization campaigns and conduct media monitoring of campaigns in Nigeria, including interviewing keys stakeholders. These interviews and activities were reported in various media.

##### Health Performance Award Ceremonies

The JAP organized 2 award ceremonies to recognize the contribution of health workers, especially supervisors and other stakeholders, for outstanding performance during polio campaigns in their communities and to reward them with certificates during award ceremonies. This has helped to improve SIA quality in areas with poor performance.

### Evaluation of What JAP Did for Polio

We evaluated the impact of JAP activities by determining the total number of media materials produced. We also assessed the number of newspaper clips and bulletins published in support of polio eradication and evaluated social data such as the number of noncompliant households that become compliant after JAP interventions in noncompliant communities. Since the JAP focused advocacy efforts on key stakeholders in identified high-risk communities to address any pending issues, we analyzed SIA data to test whether there was a relationship between media reporting through JAP activities and caregivers’ sources of information for polio immunization campaigns. Data were obtained from the Independent Monitoring Board of the Global Polio Eradication Initiative (GPEI) after every round of SIAs.

## RESULTS

During 2007–2015, >500 reports were published and aired by JAP members, which mainly portrayed immunization positively. Table 1 shows quotations from prominent religious and political leaders that were published in various newspapers in Nigeria. The deputy governor of Kaduna State, Ibrahim Yakowa, was quoted on the relevance of JAP in the *New Nigerian Weekly* of 6 December 2012 (Table 1). Prominent religious leaders were also interviewed, and reports were disseminated by the JAP. On 14 February 2013, Dr Khalid Aliyu, the secretary general of the umbrella body for Muslims in Nigeria, Jama'atul Nasrul Islam, was interviewed by the JAP, and the interview was published in *Leadership* newspaper. Sheikh Ibrahim Al Zazzaky, the leader of the Shiite movement in Nigeria, was also interviewed by the JAP, on 9 December 2013. The table also shows that an interview with another renowned Islamic scholar, Sheikh Ahmad Mahmud Gumi, was published by the JAP in *Daily Newswatch* newspaper on 24 May 2013.

**Table 1.** Quotations in Select Nigerian Newspapers on the Role of the Journalists Initiatives on Immunisation Against Polio (JAP), 2009–2013

Source	Quotation	Publication Date (Page No.)	Publication
Mrs Melissa Derry, Programme Officer, Global Health Advocacy, Bill and Melinda Gates Foundation	“I am impressed and happy to hear about …[the JAP]. It is interesting to know that journalists are directly involved in fighting against the crippling disease. It would be an incredible milestone to achieve the eradication of polio in Nigeria.”	14 Apr 2009	*Thisday*
Ibrahim Yakowa, Deputy Governor	“We want you [the JAP] to adequately sensitize the people on efficacy of polio vaccines.”	6 Dec 2012	*New Nigerian Weekly*
Alhaji Mustapha Jumare Chairman, Kaduna State Action Committee on Immunization	“Insecurity [is] impeding our efforts to eradicate polio.”^a^	13 Dec 2012 (p21)	*New Nigerian*
Sheikh Ibrahim Al Zazzaky	“I support polio immunization ….It is good that JAP is sensitizing the people.”	9 Dec 2013	*Nigerian Newsday*
Dr Khalid Aliyu, Secretary General of JNI	“[O]ur work is to look into controversial issues and come with solutions. Polio vaccine is safe.”	14 Feb 2013 (p9)	*Leadership*
Sheikh Ahmad Mahmud Gumi	“If polio must be eradicated then every child must be immunized.”	24 May 2013 (p69)	*Daily Newswatch*
Dr Abdullahi Bello G.	“The method introduce against polio by JAP is very effective.”	15 Dec 2013 (p52)	*National Mirror*

^a^ Jumare was interacting with the JAP.

Data analysis focused on number of noncompliant households that became compliant in settlements with JAP interventions. For the June 2015 round of SIAs in Kaduna, for instance, initial noncompliance formed the baseline for measuring the impact of JAP activities in the settlements. A total of 30 settlements in 5 wards of the 5 very high-risk LGAs (ie, Igabi, Ikara, Giwa, Kaduna North, and Zaria) of Kaduna State were collated. Data revealed that, of 5991 noncompliant children recorded during the June 2015 round, 5122 (85.5%) received vaccination, following the conduct of JAP activities (Table 2).

**Table 2.** Polio Immunization Status Among Children in Very High-Risk Local Government Areas (LGAs) of Kaduna State, Nigeria, Before and After Intervention by the Journalists Initiatives on Immunisation Against Polio (JAP), June 2015

LGA, Ward, Settlement	Immunization Status, Children, No.		
	Unimmunized at Baseline	Immunized After JAP Intervention	Unimmunized After JAP Intervention
Makarfi, Dandamisa			
Rugar jafaru	38	26	12
Rugar fulani alu	180	165	15
Rugan Madaki Bawa	31	15	16
Anguwar Sarki Aba Mallam	176	166	10
Anguwar Liman Aba Mallam	154	137	17
Anguwar Hamza Aba Mallam	150	113	37
Anguwar Sale Aba Mallam	120	107	13
Gidan Mota	140	105	35
Rugar Mai Kubbare	60	37	23
Audiga	110	96	14
Ikara, Kurmin Kogi			
Kurmin Kogi	281	186	95
Kurmin Kogi	69	45	24
Kurmin Kogi	268	208	60
Kurmin Kogi	152	127	25
Kurmin Kogi	88	75	13
Igabi, Rigasa			
Birnin Barwa	250	231	19
Rimaye	400	365	35
Sabon Gida Likarbu	340	312	28
Ribisa	350	344	6
Kempa	450	407	43
Zaria, Tudun Wada			
Nasarawan Kofar Doka	400	351	49
Nakwama	150	106	44
Bayan library	300	272	28
Fire service	320	262	58
Pampo layout	300	280	20
Giwa, Shika			
Salanken Sarki	289	212	77
Kurku	320	287	33
Kaduna North, Kabala			
Katsinawa Risko	33	26	7
Sayawan Lungu	42	35	7
Sayawan Akuru	30	25	5
Total	5991	5123	868

**Figure 1.**
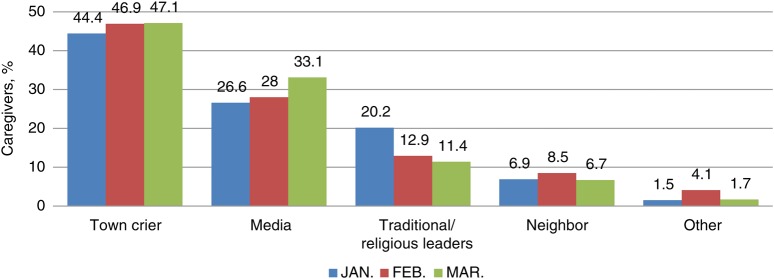
Influence of the Journalists Initiatives on Immunization Against Polio (JAP) as a source of information about supplementary immunization rounds for caregivers in Kaduna state, Nigeria during 2007 (Source: WHO Nigeria, Independent Monitoring Data).

Sources of information covering 3 rounds of SIAs in Kaduna in 2007 before and after the JAP was formed were analyzed. There was an increase in the numbers of caregivers that had heard about SIAs rounds from the media, from an initial 26% in January 2007 to 33% before March 2007. The activities of the JAP resulted in more caregivers citing media as the source of information (Figure 1). This is in contrast to the citing of traditional leaders' as sources of information, the frequency of which decreased from 20.2% in January 2007 to 14% in March of the same year. The traditional source of information, the town announcers, almost plateaued with 46.9% and 47.1% for February and March of 2007, respectively. Similarly, citation of “neighbor” and “other sources of data” decreased with the advent of the JAP.

## DISCUSSION

We found that, from 2007 when the JAP was initiated by the WHO and 8 journalists in Kaduna State, the partnership has continued to grow in strength, with changing roles for the journalists, based on current realities. From an initial presence in Kaduna State, the JAP has expanded to 10 additional states across the northwest (Sokoto, Kano, Katina, Jigawa, and Zamfara states), the northeast (Bauchi, Borno, and Gombe), and the north-central region (Niger and FCT). The national headquarters, though, remained in Kaduna.

We posit that journalists as forces of social change could alter the realities of the society which they serve. For instance, the messages in the media facilitated by the JAP were positive-endorsement stories involving influential religious and political leaders. The effect the messages evoked was predicated on the media's positive influence on the public. This argument aligned with the perspectives of different schools that have their thrust on the social responsibility role of journalists [19–22].

The JAP identified and elevated the position of religious, traditional, and political leaders to provide more insights into the benefits of polio immunization. These messages build the confidence of ordinary community members in the noncompliant communities to demand for immunization.

The program officer of global health advocacy for the Bill and Melinda Gates Foundation, Mrs Melissa Derry, while meeting with the JAP in Abuja during Mr Gates' visit in 2009, remarked, “I am impressed and happy to hear about a body called the Journalists Initiatives on Immunization Against Polio. It is interesting to know that journalists are directly involved in fighting against the crippling disease. It would be an incredible milestone to achieve the eradication of polio in Nigeria” [15].

The activities of the JAP, such as the town hall meetings, provided a common platform for representatives of noncompliant communities to share information about the importance of immunization. This was achieved through the creation of a conducive atmosphere to discuss and understand the knowledge gaps and reasons why heads of noncompliant households had resisted polio immunization. The meetings always concluded on positive notes, as previously noncompliant heads of households presented their children for immunization afterward.

The JAP helped noncompliant caregivers to realize that accepting polio vaccination was a way to prevent their children from experiencing a lifetime of being crippled. This was reinforced by the JAP's strategy of working with community and religious leaders. The JAP has therefore become a vehicle of social mobilization, education, and enlightenment, and the community members view them and the media they represent as better avenues for promoting their causes.

Despite incessant anti-OPV campaigns, the partnership with the JAP and the messages they released have created more awareness, especially among noncompliant populations, to improve immunization uptake in some traditional hardcore noncompliant communities of the north. They have become a formidable force to reckon with in the fight against wild poliovirus in northern Nigeria.

Our intervention to form a partnership with the JAP worked but has some limitations. First, JAP members are constrained from being wholly committed to the cause of polio eradication in Nigeria, owing to their primary assignments and the editorial policies of the divergent media they represent. Second, there was no systematic mechanism for data collection over the years to fully estimate the impact of JAP activities. Notwithstanding these limitations, the activities conducted by JAP have projected positive media messages to silence the untoward effect of anti-OPV campaigns orchestrated by equally prominent clerics and scholars.

The National Primary Healthcare Development Agency can engage an independent agency to conduct an in-depth survey to explore the divergent opinions of caregivers on JAP activities on the PEI, to strengthen the partnership for creating awareness on routine immunization and for future public health interventions. Before the survey, state teams with established JAP members can capitalize on the opportunity offered by positive media messages to monitor, collate, and analyze the impact of media activities with measurable indicators. It is also recommended that public recognition of the partnership with the JAP by government, WHO, UNICEF, and other GPEI partners be institutionalized to motivate members and those aspiring to be part of the association to continue with the objectives of the association. It will also encourage their employers to support them to participate more in planned activities.
